# A Comprehensive Genome-Wide Association Study of Carotenoid and Capsaicinoid Contents in *Capsicum chinense* Germplasm

**DOI:** 10.3390/ijms241813885

**Published:** 2023-09-09

**Authors:** Mesfin Haile, Nayoung Ro, Ho-Cheol Ko, Hyeonseok Oh, Gi-An Lee

**Affiliations:** National Agrobiodiversity Center, National Institute of Agricultural Sciences, Rural Development Administration, Jeonju 54874, Republic of Korea; mesfinhaile97@gmail.com (M.H.); hchko@korea.kr (H.-C.K.); zzjiy@korea.kr (H.O.); gkntl1@korea.kr (G.-A.L.)

**Keywords:** carotenoids, capsaicinoids, GBS, GWAS, SNP

## Abstract

Pepper is a highly important vegetable globally, both economically and nutritionally. However, to efficiently select and identify genetic resources for pepper breeding programs, it is crucial to understand the association between important traits and genetic factors. In this study, we investigated the genetic basis of carotenoid and capsaicinoid content in 160 *Capsicum chinense* germplasms. The study observed significant variability in carotenoid and capsaicinoid content among the germplasms. Correlation analysis revealed a strong positive correlation between violaxanthin and antheraxanthin. In contrast, capsaicin and dihydrocapsaicin displayed negative correlations with individual carotenoids but exhibited a strong positive correlation between the two compounds (r = 0.90 ***). Genotyping-by-sequencing (GBS) was performed on 160 genotypes of pepper germplasm, which identified 47,810 high-quality SNPs. A comprehensive genome-wide association analysis was performed using these SNPs to identify SNPs associated with carotenoids and capsaicinoids, revealing 193 SNPs that exhibited significant associations. Specifically, 4 SNPs were associated with violaxanthin, 2 with antheraxanthin, 86 with capsorubin, 5 with capsanthin, 63 with zeaxanthin, 3 with β-cryptoxanthin, and 2 with α-carotene. With further studies, the significantly associated SNPs identified in this study have the potential to be utilized for selecting pepper accessions with high carotenoid and capsaicinoid contents. Additionally, the genes associated with these significant SNPs will be used to understand their roles and involvement in the biosynthesis pathway of carotenoids and capsaicinoids. Understanding the function of these genes can provide insights into the molecular mechanisms underlying the production of these bioactive compounds in pepper. The findings of this study hold valuable implications for selecting pepper varieties with desirable traits and developing breeding programs aimed at enhancing the nutritional and medicinal properties of pepper.

## 1. Introduction

Pepper is a member of the nightshade (*Solanaceae*) family and the genus *Capsicum*. The genus *Capsicum* has approximately 35 species [1], five of which are domesticated and economically important: *Capsicum annum* L., *Capsicum chinese* Jacq., *Capsicum frutescens* L., *Capsicum baccatum* L., and *Capsicum pubescens* Ruiz and Pav. [2]. According to FAOSTAT [3] data from 2010 to 2021, the total production of pepper has shown a notable growth of approximately 20.28%. Within this period, green pepper production increased by 18.12%, while dry pepper production showed a significant growth of 36.43%. In the year 2021, global pepper production reached 41.13 million tons, with 36.89 million tons of fresh pepper and 4.84 million tons of dry pepper. Among the top producers in 2021, China led the world in fresh pepper production with 16.72 million tons, followed by Türkiye with 3.09 million tons and Indonesia with 2.75 million tons. In terms of dry pepper production, India emerged as the leading producer, contributing 2.05 million tons to the global market [3].

The pepper species are rich in bioactive compounds that possess a wide range of beneficial properties. These compounds are known for their potential analgesic, anti-obesity, cardioprotective, pharmacological, neurological, and dietary effects [4]. Furthermore, they exhibit significant antibiotic activity and have been found to lower serum cholesterol levels when consumed in moderate amounts as part of a regular diet [5,6,7]. Moreover, these compounds demonstrate anticarcinogenic, antifungal, antibacterial, antiviral, lipid degradation, antithrombotic, and anti-inflammatory activities [8]. Several studies, conducted both in vitro and in vivo, have demonstrated that *C. chinense* exhibits protective effects, including antioxidant and anticancer activity [9,10,11,12]. These effects suggest its potential for reducing or preventing chronic diseases [13]. The diverse array of bioactive compounds found in pepper highlights its value as an ingredient with various health benefits. Major bioactive compounds present in *Capsicum* species include carotenoids and capsaicinoids.

Carotenoids are bioactive compounds that contribute to the vibrant and diverse colors observed in pepper fruits [14,15]. *Capsicum* is known to have a high concentration of carotenoids, and the different colors of the peppers are due to their different carotenoid profiles [16]. The different colors can also affect the flavor of the peppers, with yellow, orange, and red peppers being sweeter than green peppers, and they may also be related to higher glucose content as they ripen [17]. The carotenoid composition of pepper predominantly includes capsorubin, capsanthin, β-carotene, lutein, β-cryptoxanthin, zeaxanthin, violaxanthin, and antheraxanthin [18]. The concentration of the bioactive compounds can be influenced by factors such as the amount of sunlight, soil, season, crop region, temperature changes, fruit variety, and maturity level [19,20,21]. Another important bioactive compound in pepper is capsaicinoids, which are alkaloid compounds responsible for the spiciness of chilies [22]. The two major capsaicinoids are capsaicin and dihydrocapsaicin, which comprise more than 90% of the total capsaicinoid content found in the fruit [23]. Capsaicin is a flavorless, odorless, and colorless compound present in varying amounts in different pepper varieties [24]. Capsaicinoids have significant biological activity and are relevant to various fields, including medicine, food science [25,26,27], and the defense industry [28] because of their ability to cause intense irritation and burning sensations in the eyes, nose, throat, and skin upon contact.

Molecular genetics plays a crucial role in crop improvement by utilizing molecular tools to identify DNA changes in individual plants. Among the genetic markers, single nucleotide polymorphisms (SNPs) have become the preferred choice and are extensively utilized in agricultural breeding programs [19]. To identify SNPs associated with traits of interest, genome-wide association studies (GWAS) offer a powerful approach. GWAS scans the entire genome of a population, testing for allele frequency differences in genetic variants between individuals who share common ancestry but exhibit phenotypic differences [29,30]. In major crops such as maize, rice, barley, tomato, wheat, sorghum, soybean, and watermelon species, GWAS using SNPs derived from genotyping-by-sequencing (GBS) has been widely employed [31,32,33,34,35]. This methodology has proved valuable in unraveling the genetic basis of various traits and aiding crop breeding efforts.

This study aimed to identify SNPs associated with carotenoid and capsaicinoid contents in the pepper germplasm of *Capsicum chinense*, a highly genetically diverse pepper species. The results of this study have the potential to significantly contribute to the breeding of improved pepper varieties with enhanced nutritional and medicinal properties. The use of SNP markers can accelerate the breeding process and facilitate the selection of plants with desirable traits, while the underlying genes can provide insights into the biochemical pathways involved in the biosynthesis of bioactive compounds in pepper. Further research can be conducted to explore the potential applications of these findings in pepper genomics and breeding.

## 2. Results

### 2.1. Carotenoids and Capsaicinoids Contents

The results of the descriptive analysis for ten bioactive traits (violaxanthin, antheraxanthin, capsorubin, capsanthin, zeaxanthin, β-cryptoxanthin, α-carotene, β-carotene, capsaicin, and dihydrocapsaicin) in a sample of 160 germplasms are summarized in Table 1. The table presents the mean, standard error, standard deviation, range, and count for each variable. The mean values for each variable are reported in mg/100 g units. Among the variables, capsaicin had the highest mean value of 1836.97 mg/100 g, indicating a relatively high concentration of capsaicin on average in the germplasms. On the other hand, β-cryptoxanthin had the lowest mean value of 8.37 mg/100 g. The range values indicate the difference between the highest and lowest values observed for each variable in the sample, also presented in mg/100 g units. Capsaicin exhibited the widest range of values, ranging from 0 to 11,632.93 mg/100 g, indicating significant variability among the germplasms in terms of capsaicin content. In contrast, β-cryptoxanthin had the narrowest range, spanning from 0 to 81.81 mg/100 g. The substantial range values observed for carotenoids and capsaicinoids further indicate considerable variability among the germplasms for these particular traits. This variation could have implications for breeding programs and genetic studies aiming to develop pepper varieties with the desired levels of carotenoids and capsaicinoids.

### 2.2. Correlation Analysis

**Figure 1:**
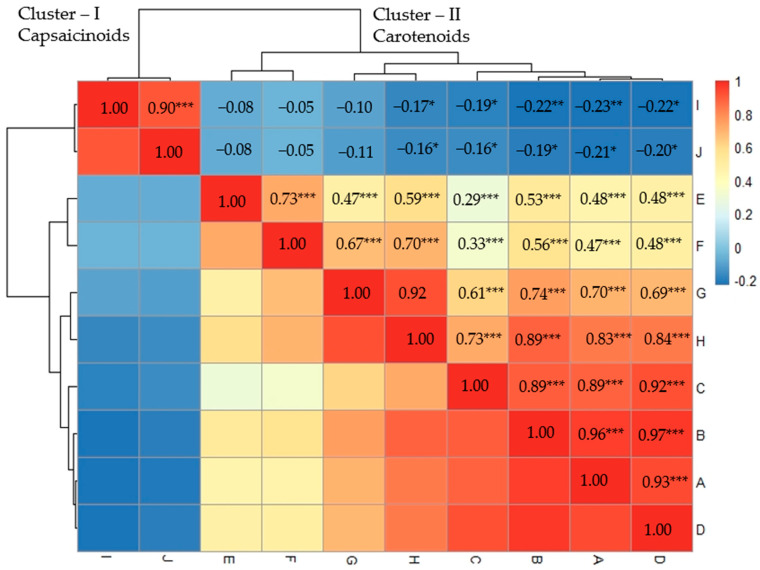
The heatmap correlation of carotenoids and capsaicinoids in 160 *C. chinense* germplasms. The heatmap displays Pearson’s correlation values, with colors indicating the strength and direction of the correlations, located on the right side of the picture. The carotenoids and capsaicinoids are labeled as follows: A: violaxanthin, B: antheraxanthin, C: capsorubin, D: capsanthin, E: zeaxanthin, F: β-cryptoxanthin, G: α-carotene, and H: β-carotene. The capsaicinoids are represented by I: capsaicin and J: dihydrocapsaicin. Significance levels are indicated with *, **, and *** for *p* < 0.05, *p* < 0.01, and *p* < 0.001, respectively.

The present study used correlation analysis to investigate the pairwise associations among 10 variables labeled “A” through “J” (Figure 1). The correlation matrix showed that antheraxanthin and capsanthin had a strong positive correlation (r = 0.97 ***), and violaxanthin and antheraxanthin had a strong positive correlation as well (r = 0.96 ***). This suggests that changes in one variable may correspond with changes in the other variable in a predictable manner. Capsorubin and capsanthin had a strong positive correlation (r = 0.92 ***). On the other hand, Zeaxanthin and β-cryptoxanthin showed a moderate positive correlation (r = 0.73 ***). In relation to carotenoids, the weakest correlations were observed between capsorubin and zeaxanthin (r = 0.29 ***), and subsequently with β-cryptoxanthin (r = 0.33 ***). α-Carotene and β-carotene had a strong positive correlation (r = 0.92), indicating a potentially meaningful relationship between these variables. There was a strong positive correlation between capsaicin and dihydrocapsaicin (r = 0.90). Contrarily, capsaicin and dihydrocapsaicin showed negative correlations with individual carotenoids. Figure 1 visually depicts the clear clustering of capsaicinoids (cluster-I) and carotenoids (cluster-II), indicating a distinct relationship between these variables. Additionally, the negative correlations within the dataset ranged from 0.05 to 0.23.

### 2.3. Genotyping-by-Sequencing

**Figure 2:**
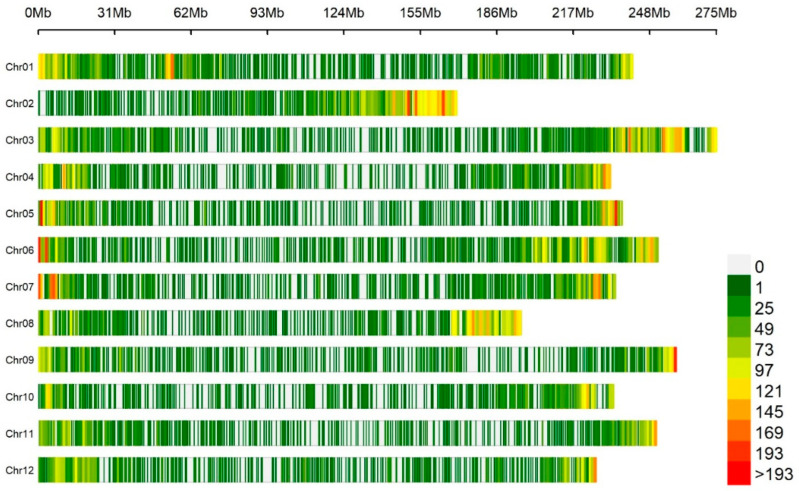
The distribution patterns of SNPs across all 12 chromosomes from 160 *C. chinense* accessions are illustrated using a 1 Mb window size. The heatmap colors in the figure visually represent the density of the SNPs, offering insights into their distribution patterns.

GBS was performed on 160 pepper germplasms using the Illumina HiSeq X Ten platform, resulting in a substantial dataset comprising approximately 1.2 billion total trimmed reads. The average mapping depth for a single accession was determined to be 17.61×, indicating a robust and thorough coverage of the pepper genome. After the variant calling process, a comprehensive set of 1,859,683 single nucleotide polymorphisms (SNPs) was identified, encompassing all 12 pepper chromosomes. The sequencing statistics for 160 *C. chinense* accessions are provided in Supplementary Table S1. To ensure the integrity and reliability of the data, a stringent filtering approach was applied. SNPs with a minor allele frequency below 5% and those exhibiting missing data exceeding 30% were excluded and obtained 47,810 high-quality SNPs for further analysis. Figure 2 illustrates the distribution patterns of SNPs across the 12 chromosomes of 160 *C. chinense* accessions. A window size of 1 Mb was used for this analysis. The heatmap colors in the figure depict the density of SNPs, providing a visual representation of their distribution patterns. These SNPs were used for genetic association studies to explore the genetic basis of important pepper bioactive compounds (carotenoids and capsaicinoids).

### 2.4. Genome-Wide Association Analysis

**Figure 3:**
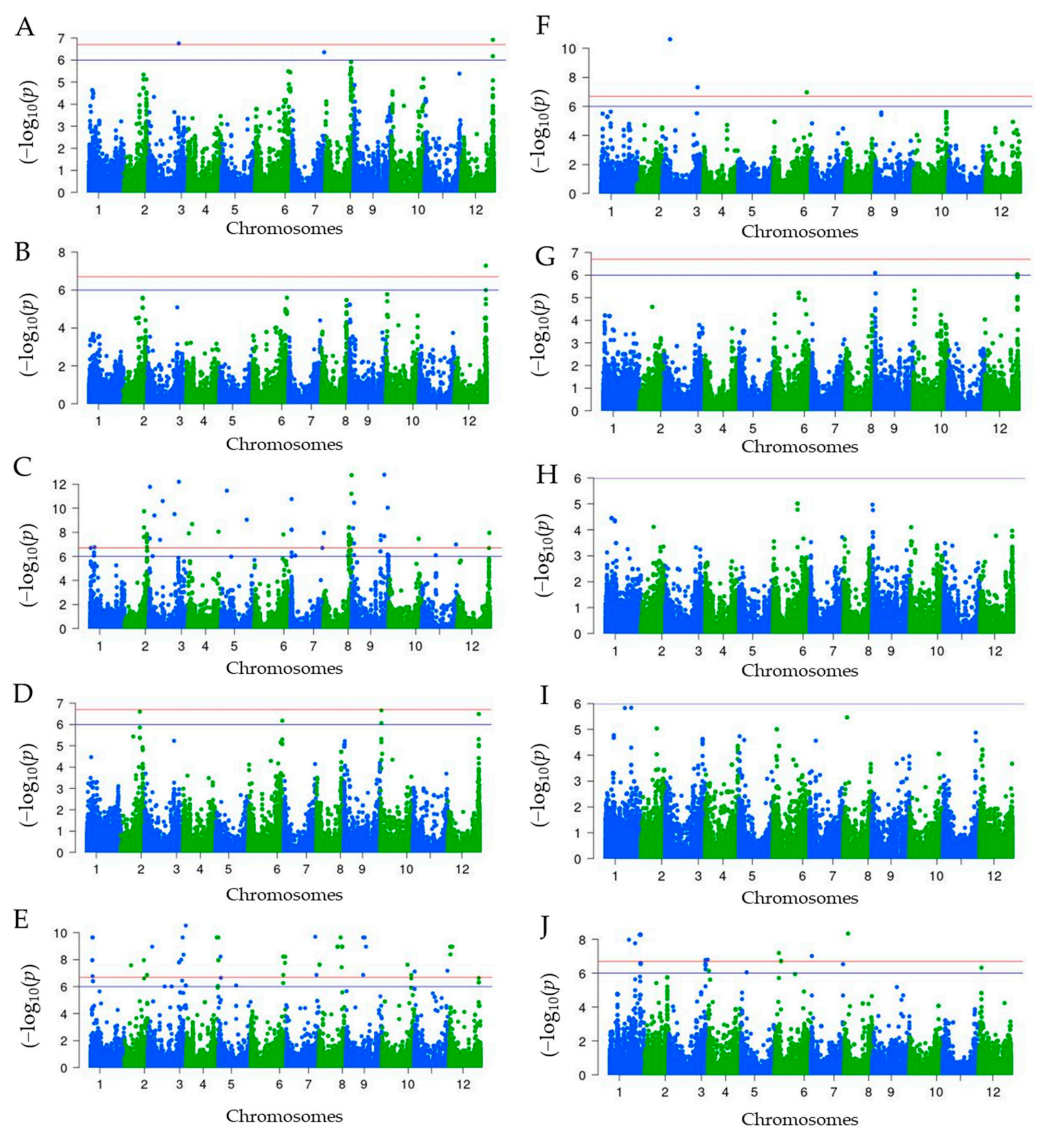
Manhattan plots illustrating the association between carotenoids ((**A**): violaxanthin, (**B**): antheraxanthin, (**C**): capsorubin, (**D**): capsanthin, (**E**): zeaxanthin, (**F**): β-cryptoxanthin, (**G**): α-carotene and (**H**): β-carotene) and capsaicinoids ((**I**): capsaicin and (**J**): dihydrocapsaicin) using 160 *C. chinense* accessions. Each dot in the plot represents a single SNP, where the x-axis denotes the genomic location, with chromosomes colored and labeled accordingly. The y-axis represents the association level, measured as −log_10_(*p*). The blue line (−log_10_(*p*) = 6) corresponds to a significance threshold of *p* < 0.05, while the red line (−log_10_(*p*) = 6.7) represents a more stringent significance threshold of *p* < 0.01. The horizontal blue and red lines indicate the Benferroni-corrected significance thresholds for the association of SNPs with traits.

**Figure 4:**
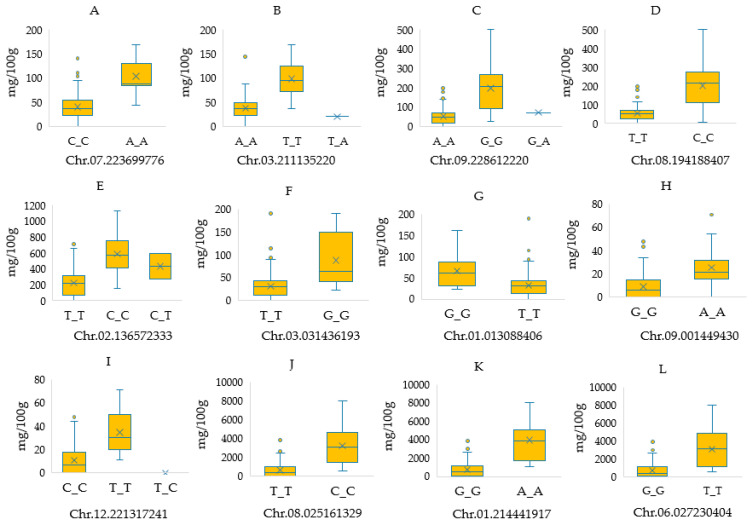
Box plots illustrating the allelic effect of selected SNP markers significantly associated with various chemical traits. The chemical traits depicted include violaxanthin (**A**,**B**), capsorubin (**C**,**D**), capsanthin (**E**), zeaxanthin (**F**,**G**), α-carotene (**H**,**I**), and dihydrocapsaicin (**J**–**L**). The x-axis represents the alleles found at specific chromosomes and positions, while the y-axis represents the average values of the chemical traits.

The genome-wide association analysis conducted using 47,810 SNPs aimed to identify SNPs associated with carotenoids and capsaicinoids. The results of the analysis were visualized in Manhattan plots (Figure 3) and quantile-quantile (Q-Q) plots (Supplementary Figure S1). In total, 193 SNPs exhibited significant associations with carotenoids and capsaicinoids. Among these, 165 SNPs were found to be associated with individual carotenoids (β-carotene), while 28 SNPs were associated with capsaicinoids, specifically dihydrocapsaicin. Regarding carotenoids, the analysis revealed a diverse set of SNPs that were significantly associated. Violaxanthin showed an association with 4 SNPs, antheraxanthin with 2 SNPs, capsorubin with 86 SNPs, capsanthin with 5 SNPs, zeaxanthin with 63 SNPs, β-cryptoxanthin with 3 SNPs, and α-carotene with 2 SNPs. In Figure 4, the box plots display the allelic frequency of selected SNP markers that exhibit significant associations with specific chemical traits. The depicted chemical traits include violaxanthin (A, B), capsorubin (C, D), capsanthin (E), zeaxanthin (F, G), α-carotene (H, I), and dihydrocapsaicin (J, K, L).

Of the SNPs associated with violaxanthin, two were identified on chromosome 12. The first SNP (T/C allele, 221,105,042 bp) was found in an intergenic region, while the second SNP (A/G allele, 221,085,307 bp) was located within the gene encoding 6,7-dimethyl-8-ribityllumazine synthase. Another significant association was observed with a SNP on chromosome 3 (A/T allele, 211,135,220 bp), which resides within the gene encoding 4-hydroxycinnamoyl-CoA ligase 2. Additionally, one SNP on chromosome 07 (223,699,776 bp) was also found to be significantly associated with violaxanthin. Furthermore, two SNPs located on chromosome 12 demonstrated significant associations with both violaxanthin and antheraxanthin in relation to capsorubin, a total of 86 SNPs showed significant association, surpassing a Benferroni-corrected threshold of −log_10_(*p*-value) = 6.0. Out of these, 57 SNPs also exceeded a higher threshold of −log_10_(*p*-value) = 6.7. The SNPs that exhibited significant association with capsorubin were distributed across various chromosomes. Notably, the highest number of associations was observed on chromosomes 8, 9, and 2, with 24, 16, and 14 SNPs, respectively.

Table 2 presents a list of 24 highly significant SNPs associated with capsorubin in *C. chinense.* The characteristics of the gene (name, function) in which significant SNPs were identified can be found in Supplementary Table S2. Out of these, 14 SNPs were located within genic regions, while the remaining 10 were found in intergenic regions. These genic SNPs were found in several genes, including NADP-malic enzyme 3, vacuolar protein sorting protein, and GTP-binding protein. Additionally, several SNPs were linked to proteins with unknown functions that were detected in the study.

In relation to capsanthin, β-cryptoxanthin, and α-carotene, a total of 10 SNPs were identified, with eight SNPs located in genic regions and three SNPs located in intergenic regions. Among these SNPs, five were associated with capsanthin, with three situated within genic regions and two found in intergenic regions. The genic SNP was specifically located within the CA12g19380 gene, which encodes for 6,7-dimethyl-8-ribityllumazine synthase. For β-cryptoxanthin, three SNPs were identified, with one SNP located in an intergenic region. In the case of α-carotene, two SNPs were found, both residing within genic regions. One genic SNP was located within the CA00g41080 gene, which encodes for RAB1X, a protein involved in intracellular transport. The other genic SNP was located within the CA12g19190 gene, which encodes for ubiquitin carboxyl-terminal hydrolase 19-like. A significant association with zeaxanthin was observed for a total of 63 SNPs. Among these, the major SNPs were distributed across chromosomes 3, 8, 12, and 1, with 12, 9, 7, and 7 SNPs, respectively. Among the identified SNPs associated with zeaxanthin, a total of 21 were selected and presented in Table 2. Out of these 21 SNPs, 12 were located within genic regions, and 9 were found in intergenic regions. The genic SNPs were found to be associated with various gene functions, including histone deacetylase, DNA binding protein, coatomer alpha subunit, STY-L protein, transcription factor BIM1, photosystem II processing protein, chaperone regulator, mitogen-activated protein kinase, and sinapyl alcohol dehydrogenase-like 3.

For capsaicinoids, a total of 28 significant SNPs were found to be associated with dihydrocapsaicin, while no SNP reached the significant threshold of −log_10_(*p*-value) = 6.0 for capsaicin. Out of the significant SNPs linked to dihydrocapsaicin, 15 SNPs exceeded the highly significant threshold of −log_10_(*p*-value) = 6.7. These SNPs associated with dihydrocapsaicin were distributed across different chromosomes, including Chr08 (T/C, 25,161,329 bp), Chr01 (A/G, 208,666,749 bp), Chr01 (A/T, 209,615,478 bp), Chr01 (A/G, 214,441,815 bp), Chr01 (A/G, 214,441,917 bp), Chr01 (C/T, 214,442,584 bp), Chr01 (C/T, 132,753,120 bp), Chr01 (A/C, 176,184,702 bp), Chr06 (G/T, 27,230,404 bp), and Chr07 (A/C, 6,571,083 bp). Several of these SNPs were located within genes encoding various proteins, such as ATP binding protein, late embryogenesis abundant protein (LEA) family protein, steroleosin-B, and small MutS-related (Smr). Additionally, SNPs were also identified in intergenic regions, which are regions that do not correspond to known genes.

### 2.5. SNP Markers Showing Pleiotropic Effects

The common SNPs associated with multiple traits are presented in Table 3. These SNPs were identified across different chromosomes, residing either within genic or intergenic regions. Among the traits examined, violaxanthin, antheraxanthin, capsorubin, and capsanthin were found to have common SNPs. Chromosomes 2, 3, 7, and 12 contained the SNPs linked to these traits. Notably, some of these SNPs were located within genes responsible for encoding specific proteins, such as 6,7-dimethyl-8-ribityllumazine synthase and 4-hydroxycinnamoyl-CoA ligase 2. However, in other instances, the SNPs were detected in intergenic regions.

## 3. Discussion

### 3.1. Carotenoids and Capsaicinoids Contents

Peppers’ widespread popularity stems from their visually appealing colors, diverse flavors, culinary versatility, and nutritional benefits. Peppers contain a variety of nutrients and bioactive compounds, including vitamins, carotenoids, capsaicinoids, anthocyanins, phenolic acids, and flavonoids [36,37]. Carotenoids in peppers are of particular interest among these components because they contain provitamin A carotenoid (β-carotene, α-carotene, and β-cryptoxanthin) as well as other carotenoids that are crucial for maintaining human eye health (lutein, and zeaxanthin) [37]. Carotenoids have nutritional value, but they also function as antioxidants by quenching reactive oxygen species and neutralizing free radicals because they contain conjugated double bonds. These carotenoids have been linked to a lower risk of developing some chronic medical conditions [38]. In this experiment, eight carotenoids were quantified using 160 pepper accessions. The highest total carotenoid content was 2426.49 mg/100 g DW. Capsanthin was the highest individual carotenoid and contributed more to the total carotenoid content. A similar report indicated that among the nine carotenoids from the sweet red pepper variety that were quantified, capsanthin was the one with the highest concentration [37]. The pepper accessions showed a wider variation in carotenoid content, ranging from 4.36 to 2426.49 mg/100 (Table 1). These variations in genotypes determine that specific carotenoid biosynthetic enzymes have been linked to a significant variation in carotenoid profiles among *Capsicum* species and cultivars [39].

Capsaicinoids, a category of alkaloids responsible for the hot or spicy flavor, are synthesized and accumulated by the plant and are found largely in placental tissue near the seeds [40]. Their concentration is determined by genotype, fruit maturity, and growing circumstances [41]. The word “capsaicinoids” refers to a class of pungent chemical mimics found only in chili peppers [42]. Capsaicinoids have anticarcinogenic effects that inhibit the androgen-dependent growth of breast cancer, colon cancer, prostate cancer, and stomach adenocarcinomas [43,44,45]. We measured the content of capsaicinoids (capsaicin, dihydrocapsaicin, capsiate, and dihydrocapsiate) in pepper accessions and found that capsaicin and dihydrocapsaicin were the two major capsaicinoids with the highest amounts (Table 1). Due to the limited presence of capsiate and dihydrocapsiate in only a few accessions, we excluded the data for these two compounds from the analysis. This finding is in agreement with a report that states the most abundant and potent capsaicinoids in peppers (and consequently pepper extracts) are capsaicin and dihydrocapsaicin [44,46]. Capsaicinoid content varied greatly among pepper accessions, with total capsaicinoid content ranging from 0 to 19,671.1 mg/100 g DW (Table 1).

### 3.2. SNPs Associated to Carotenoids and Capsaicinoids

The impact of genetic variation on the biochemical components of plant species has been explored using a number of molecular biology approaches. GWAS analysis plays a critical role in identifying genetic markers associated with traits of interest. A marker-trait association study was carried out utilizing 47,810 high-quality SNPs derived from 160 pepper accessions. A total of 193 SNPs were identified as significantly associated with individual carotenoids (165 SNPs) and capsaicinoids (28 SNPs). The SNPs discovered in this study were located in both genic and intergenic regions. SNPs can occur at different frequencies in different regions of chromosomes, including coding sequences of genes, non-coding regions of genes, and intergenic regions between genes [47,48]. The presence of pleiotropy suggests that there could be shared genetic factors among related traits [49]. SNPs that exhibited associations with multiple traits, specifically the individual carotenoids, are presented in Table 3. Similarly, in another study conducted on *Cucurbita maxima* Duchesne, SNP markers showing pleiotropic effects among the different analyzed carotenoids were reported [50].

The present study identified a SNP on chromosome 09 associated with the carotenoid capsorubin within a gene encoding an auxin response factor (ARF). This finding aligns with the existing literature that emphasizes the involvement of ARFs in plant growth and development, as well as their potential roles in carotenoid biosynthesis and responses to abiotic stresses [51]. Research on the role of auxin in the growth, development, and stress response of the model plant Arabidopsis thaliana and major field crops like wheat, maize, and rice, among others, has been comprehensive and extensive [52,53,54,55,56]. ARFs are a family of transcription factors that regulate the expression of genes in response to auxin and have been implicated in various physiological processes in plants [39]. Previous studies have demonstrated their importance in regulating plant growth, development, and hormone signaling pathways, particularly in response to auxin [57,58,59,60,61,62,63]. However, their specific roles in carotenoid biosynthesis and abiotic stress responses have been less explored until recently. The findings of the previous study [51] contributed to our understanding of the functional relevance of ARFs in carotenoid metabolism and stress tolerance. By analyzing the sweet potato IbARF5 gene, the researchers observed and confirmed an increase in carotenoid contents and enhanced tolerance to salt and drought in transgenic Arabidopsis plants [51]. This study reports for the first time that the IbARF5 gene plays a vital role in regulating carotenoid biosynthesis and influencing the plant’s response to abiotic stresses. Similarly, our finding of SNPs associated with carotenoids can strengthen the previous findings and emphasize the need for further study for better understanding.

Another SNP was found on chromosome 04, which showed a significant association with zeaxanthin, one of the individual carotenoids. This SNP is located within a gene that encodes a member of the helix–loop–helix (bHLH) transcription factor (TFs) family. The bHLH is one of the largest TFs and plays a crucial role in regulating plant growth and development by interacting with other TFs in various biological processes [64,65]. Existing literature provides support for the involvement of bHLH transcription factors in the regulation of both carotenoid and capsaicinoid biosynthesis. Existing literature provides support for the involvement of bHLH transcription factors in the regulation of both carotenoid and capsaicinoid biosynthesis. A GWAS was conducted in *Capsicum* and the expression profiles of specific bHLH transcription factors, such as *CabHLH009, CabHLH032, CabHLH048, CabHLH095*, and *CabHLH100* from clusters C1, C2, C3, and C4, showed a correlation with the accumulation of carotenoids, including zeaxanthin, in the pericarp [66]. Additionally, the expression profiles of CabHLHs in clusters L5, L6, L8, and L9 were found to be consistent with capsaicinoid biosynthesis [66]. In another study, it was also noted that the regulation of broccoli carotenoid biosynthesis primarily involved the NAC, bHLH, bZip, MYB, and ERF families of transcription factors [67]. Moreover, the results obtained from the analysis of TFs gene ontology (GO) categories revealed that genes such as *bHLH66*, *PIF4*, *LOB13*, *NAC92*, and *APL* were found to be enriched in multiple categories related to chlorophyll biosynthesis, regulation of chlorophyll biosynthesis, and carotenoid biosynthetic process [67]. These findings suggest that the identified bHLH transcription factors may play crucial roles in the biosynthesis of both carotenoids and capsaicinoids. Therefore, the association of the SNP on chromosome 04 with zeaxanthin likely involves the regulation of carotenoid biosynthesis through the modulation of the corresponding bHLH transcription factor. Further investigations are important to elucidate the precise mechanisms by which these candidate bHLH transcription factors regulate carotenoid and capsaicinoid biosynthesis and to validate the functional impact of the identified SNP on zeaxanthin levels.

The present study identified 28 SNPs associated with dihydrocapsaicin, among which one SNP was found in the gene encoding an ATP-binding protein. ATP-binding proteins are recognized for their critical roles in various cellular processes in plants, encompassing energy metabolism, signaling pathways, transport processes, and enzymatic activities [68]. Additionally, the presence of SNPs in genes encoding other proteins, including histidyl-tRNA synthetase, putative LEA family protein, steroleosin-B, small MutS-related domain-containing protein, ubiquitin-activating enzyme E1c, putative serine-threonine protein kinase, putative catalytic, and putative translation elongation factor EF1A protein, emphasizes the significance of further investigation and understanding to explore their potential involvement in relevant biological processes. In addition, significant associations were observed between SNPs and carotenoid and capsaicinoid content in peppers within genes encoding proteins of unknown function. These findings emphasize the need for further investigation to uncover the roles of these genes in carotenoid and capsaicinoid metabolism, potentially involving novel enzymatic activities or regulatory functions. Functional characterization experiments, such as gene expression analysis and targeted assays, are warranted to elucidate the roles of these proteins. Comparative genomics and integrative omics approaches hold promise for gaining insights into the conservation and potential functional domains of these unknown proteins.

In conclusion, the development of improved plant varieties with enhanced bioactive compounds and nutritional values is a crucial goal in plant breeding. Marker-assisted plant breeding approaches offer a valuable tool by enabling the study of genetic variants and their association with important traits of interest. In this study, we have identified a large number of significantly associated SNPs with carotenoids and capsaicinoids. These findings can assist future studies aiming to identify potential markers for the selection of pepper germplasm with high carotenoid and capsaicinoid content. Furthermore, the genes associated with the identified SNPs will provide valuable insights into their functions and their involvement in the biosynthesis pathways of these bioactive compounds for future studies.

## 4. Materials and Methods

### 4.1. Chemicals and Plant Material

In this study, analytical grade reagents, extraction solvents, and carotenoid and capsaicinoid standards were used. The chemicals used were sourced from Sigma-Aldrich (Saint Louis, MI, USA) and included carotenoid standards such as capsanthin, capsorubin, antheraxanthin, violaxanthin, zeaxanthin, beta-cryptoxanthin, alpha-carotene, and beta-carotene, as well as capsaicinoid standards including capsaicin and dihydrocapsaicin. Additional chemicals used in the study were potassium hydroxide, dichloromethane, methanol, sodium chloride, ascorbic acid, ammonium acetate, and methyl tert-butyl ether.

The *Capsicum chinense* genetic materials used in the study consisted of 160 accessions sourced from the National Agrobiodiversity Center’s (NAC) gene bank under the Rural Development Administration (RDA) in Jeonju, Republic of Korea. Each accession was represented by ten to twelve pepper plants, with three replications, which were cultivated in NAC greenhouses following the RDA’s pepper growing methods. Fully matured pepper fruits were collected, freeze-dried, powdered, and stored in a deep freezer at −70 °C for further analysis. Detailed information about the IT (introduction) numbers and origins of the 160 *C. chinense* pepper materials can be found in Supplementary Table S3.

### 4.2. Analysis of Carotenoids

The pepper samples used in this study were all freeze-dried and powdered. The extraction, separation, and measurement of carotenoids by High-Performance Liquid Chromatography (HPLC) were conducted with minor modifications, following the procedure outlined by Kim et al. [69]. To extract carotenoids, 0.05 g of pepper powder that had been sieved through a 0.7 mm sieve was mixed with 3 mL of ethanol containing 0.1% ascorbic acid (*w*/*v*). The mixture was vortexed for 20 s and then placed in an 85 °C water bath for 5 min. Subsequently, saponification of the extract was carried out for 10 min in an 85 °C water bath using potassium hydroxide (120 L, 80% *w*/*v*). After saponification, the samples were immediately cooled on ice, and 1.5 mL of cold deionized water was added. The extraction process was repeated twice using 1.5 mL of hexane. The resulting extracts were then centrifuged at 12,009× *g*, and the supernatant was filtered through a 0.2 µm syringe filter to obtain the final carotenoid extract for analysis.

The separation of carotenoids was carried out using HPLC on an Agilent 1260/90 Infinity II system (Santa Clara, CA, USA) equipped with a C30 YMC column (250 × 4.6 mm, 3 µm; Waters Corporation, Milford, MA, USA). The detection of carotenoids was performed at a wavelength of 450 nm. The mobile phase consisted of two solvents: Solvent A, a mixture of methanol and water (92:8 *v*/*v*) containing 10 mM ammonium acetate, and Solvent B, which was 100% methyl tert-butyl ether. The carotenoids were separated using HPLC (Agilent 1260/90 Infinity II, Santa Clara, CA, USA) on a C30 YMC column (250 × 4.6 mm, 3 µm; Waters Corporation, Milford, MA, USA) and detected at 450 nm. Solvent A was a mixture of methanol and water (92:8 *v*/*v*), containing 10 mM ammonium acetate. Solvent B was made of 100% methyl tert-butyl ether. The following gradient elution conditions were employed: 0 min (83% A and 17% B), 23 min (70% A and 30% B), 29 min (59% A and 41% B), 35 min (30% A and 70% B), 40 min (30% A and 70% B), 44 min (83% A and 17% B), and 55 min (83% A and 17% B) at a 1 mL/min flow rate. Calibration curves were created for quantification purposes by graphing four distinct concentrations of carotenoid standards based on the peak area ratios of the standards. The analysis was executed in triplicate.

### 4.3. Analysis of Capsaicinoids

To prepare the sample for analysis, the freeze-dried pepper powder sample was combined with acetonitrile in a ratio of 1:10. The mixture was sonicated for 1 h to facilitate the extraction of the desired compounds. Following sonication, the sample was heated in a water bath at 80 °C for 4 h to further promote extraction. After the heating step, the mixture was subjected to centrifugation at 4 °C and 15,000 rpm for 15 min using a centrifuge. The resulting supernatant, which contains the extracted compounds, was then filtered using a Polyvinylidene fluoride (PVDF) 0.2 μm syringe filter to remove any remaining particulate matter and prepared for analysis.

The capsaicinoid analysis was conducted using Ultra-Performance Liquid Chromatography (UPLC) with an AQUITY UPLC H-Class instrument (Waters; Milford, MA, USA) and an ACQUITY UPLC HSS T3 1.8 µm 2.1 × 50 mm column. The column temperature was maintained at 30 °C, and a flow rate of 0.4 mL/min was used for the mobile phase. The sample temperature was set at 20 °C. The total run time for the analysis was 7 min, with a delay time of 5 min. An injection volume of 2 µL was used. The mobile phase consisted of a 45% acetonitrile isocratic condition. The seal solvent, purge solvent, and needle solvent were all 45% acetonitrile. The Photo Diode Array (PDA) detector was set to measure the absorbance at a wavelength of 280 nm, which is suitable for detecting capsaicinoids. To prepare the capsaicin and dihydrocapsaicin standards for analysis, precise measurements were taken. Initially, 5 mg of each standard was accurately weighed and dissolved in 5 mL of acetonitrile, resulting in a working solution with a concentration of 1000 mg/L. Working solutions for capsaicin and dihydrocapsaicin were prepared in a concentration range from 1.95 mg/L to 1000 mg/L and 1.95 mg/L to 250 mg/L, respectively. These prepared standards were then utilized in the subsequent analysis to determine the concentration of capsaicinoids present in the sample. The analysis was executed in triplicate.

### 4.4. DNA Extraction and Genotype-by-Sequencing

For the extraction of genomic DNA from the young leaves of each accession, we followed the cetyltrimethylammonium bromide (CTAB) protocol [70]. The DNA quantity was measured using the Quant-iT PicoGreen dsDNA Assay Kit from Molecular Probes (Eugene, OR, USA), and the measurement was conducted using the Synergy HTX Multi-Mode Reader from Biotek (Winooski, TV, USA). The DNA concentration was then adjusted to 12.5 ng/μL. Following that, the DNA underwent digestion using the ApeKI enzyme from New England Biolab, with a digestion time of 3 h at a temperature of 75 °C. GBS libraries were constructed following the methods previously described [71,72], with minor modifications. The GBS libraries were sequenced on the Illumina HiSeq X Ten (Illumina, Inc., San Diego, CA, USA) using paired-end reads of 151 base pairs (bp).

Demultiplexing was performed using barcode sequences, followed by removal of adapter sequences and sequence quality trimming. Adapter and barcode sequences were removed using the software Cutadapt (version 1.8.3) [73]. Low-quality sequences were eliminated using the DynamicTrim and LengthSort programs from the SolexaQA package (v.1.13) [74]. For DynamicTrim, a minimum Phred score of 20 was used as the threshold. In the case of LengthSort, short reads with a minimum length of 25 bp were retained. The BWA (Burrows-Wheeler Aligner, ver. 0.6.1-r104) [66] software was utilized to generate clean reads, which underwent preprocessing and were subsequently mapped to the *C. chinense* reference genome v1.2 (http://peppergenome.snu.ac.kr/, accessed on 9 June 2020).

### 4.5. SNP Calling and Filtering

During the mapping process, a SAM file was generated with default parameter values, except for specific options set as follows: a seed length (−l) of 30, maximum differences allowed in the seed (−k) of 1, number of threads (−t) used for processing of 16, mismatch penalty (−M) of 6, gap opening penalty (−O) of 15, and gap extension penalty (−E) of 8. The resulting SAM files were used for raw SNP discovery using SAMtools (version 0.1.16) [75]. From the obtained data, consensus sequences were extracted to determine the consensus sequences and identify any genetic variations present.

Prior to SNP detection, SNP validation was carried out using the SEEDERS [76] in-house script. Raw SNP detection was then performed, utilizing default parameter values, except for specific options set as follows: a minimum mapping quality for SNPs (−Q) of 30, a minimum mapping quality for gaps (−q) of 15, a minimum read depth (−d) of 3, a minimum InDel score for nearby SNP filtering (−G) of 30, SNPs within a certain distance (INT bp) around a gap to be filtered (−w) of 15, a window size for filtering dense SNPs (−W) of 30, and a maximum read depth (−D) of 165. The resulting SNP matrix was categorized into three groups based on the read depth: homozygous SNPs (SNP read depth ≥ 90%), heterozygous SNPs (40% ≤ SNP read depth ≤ 60%), and other SNPs. Following the filtering process, a total of 47,810 high-quality SNPs were obtained, meeting the criteria of having missing data of less than 30% and a minor allele frequency greater than 5%. These high-quality SNPs were selected for further association analysis.

### 4.6. Genome-Wide Association Analysis

The association analysis was conducted using the 47,810 SNPs obtained from 160 pepper individuals. Imputation of missing genotypes was performed using the BEAGLE algorithm [77]. For the association analysis, QTLmax 3.0 (Katy, TX, USA) [78] was utilized, employing a Linear Mixed Model (LMM) to account for population structure and relatedness among individuals. The significance threshold after Bonferroni correction was determined to be greater than 6.0, calculated as −log(0.05/47,810). Additionally, a more stringent threshold of greater than 6.7 was used, calculated as −log(0.01/47,810). These thresholds were applied to determine the level of significance for the association analysis results.

A Basic Local Alignment Search Tool (BLAST) was employed to search for candidate genes adjacent to the SNP of interest. The *Capsicum* genome database (http://peppergenome.snu.ac.kr, *Capsicum annuum CM334 (v1.6) CDS*), tomato and potato genomes at https://solgenomics.net/ and the NCBI (National Center for Biotechnology Information) database were utilized in this analysis. The search focused on a 200-kb region surrounding the SNP, including 100 kb on each side. The flanking sequences of the SNP were obtained from the *C. chinense* genome database. These sequences were then compared against the Capsicum genome database and the NCBI database to identify genes or gene regions that exhibited similarity or alignment.

### 4.7. Statistical Analysis

The Microsoft Excel program was utilized for data summary and descriptive statistics of the carotenoids and capsaicinoids. Furthermore, the R software (version 4.2.1) was employed to perform correlation analysis (“pheatmap” package).

## Figures and Tables

**Table 1:** The descriptive statistical summary of carotenoids and capsaicinoid content in 160 *C. chinense* genetic resources.

Traits	Range (mg/100 g)	Mean (mg/100 g)	SE	SD	Count
Violaxanthin	0–168.89	43.91	2.61	33.01	160
Antheraxanthin	0–445.35	127.53	7.64	96.61	160
Capsorubin	0–501.4	76.53	6.50	82.24	160
Capsanthin	0–1120.95	299.92	18.31	231.59	160
Zeaxanthin	0–190.36	34.45	2.47	31.24	160
β-Cryptoxanthin	0–81.81	8.37	0.85	10.71	160
α-Carotene	0–70.87	12.40	1.06	13.45	160
β-Carotene	0–184.33	54.09	2.96	37.42	160
Capsaicin	0–11,632.93	1836.97	158.68	2007.22	160
Dihydrocapsaicin	0–8038.17	892.58	91.01	1151.15	160

Note: Values in the table are provided in mg/100 g units. SE: standard error, SD: Standard deviation.

**Table 2:** List of selected significantly associated SNPs with carotenoids and capsaicinoids in pepper (*C. chinense*) genetic resources.

Traits	Chr.	Reference Allele	Alternate Allele	Position (bp.)	*p*.Value	−Log_10_ (*p*.Value)	Feature	Minor Allele	Major Allele
Violaxanthin	12	G	A	221,085,307	1.21 × 10^−7^	6.92	Genic	A	G
	03	T	A	211,135,220	1.77 × 10^−7^	6.75	Genic	T	A
	07	A	C	223,699,776	4.43 × 10^−7^	6.35	Intergenic	A	C
	12	C	T	221,105,042	6.71 × 10^−7^	6.17	Intergenic	T	C
Antheraxanthin	12	G	A	221,085,307	5.24 × 10^−8^	7.28	Genic	A	G
	12	C	T	221,105,042	1.02 × 10^−6^	6.00	Intergenic	T	C
Capsorubin	09	G	A	228,612,220	1.65 × 10^−13^	12.78	Intergenic	G	A
	08	C	T	194,188,407	1.77 × 10^−13^	12.75	Genic	C	T
	03	T	A	211,135,220	6.32 × 10^−13^	12.20	Genic	T	A
	03	C	T	7,882,123	1.67 × 10^−12^	11.78	Genic	C	T
	05	G	G	41,033,949	3.47 × 10^−12^	11.46	Genic	G	C
	08	C	A	193,486,347	6.22 × 10^−12^	11.21	Intergenic	C	A
	07	C	A	7,431,933	1.73 × 10^−11^	10.76	Genic	A	C
	03	A	G	97,513,286	2.51 × 10^−11^	10.60	Intergenic	A	G
	09	C	T	16,302,649	3.53 × 10^−11^	10.45	Intergenic	C	T
	09	G	C	252,016,648	9.07 × 10^−11^	10.04	Genic	G	C
	02	C	T	136,572,333	1.79 × 10^−10^	9.75	Genic	C	T
	03	A	G	180,540,684	3.08 × 10^−10^	9.51	Intergenic	A	G
	03	T	A	39,034,826	4.04 × 10^−10^	9.39	Genic	T	A
	05	T	C	180,424,789	9.00 × 10^−10^	9.05	Genic	T	C
	04	A	G	29,271,696	2.06 × 10^−9^	8.69	Genic	A	G
	08	A	G	176,546,501	3.91 × 10^−9^	8.41	Genic	A	G
	08	A	G	176,546,659	3.91 × 10^−9^	8.41	Genic	A	G
	02	A	G	136,573,788	3.94 × 10^−9^	8.40	Intergenic	A	G
	09	T	C	10,001,201	4.58 × 10^−9^	8.34	Genic	T	C
	07	C	T	7,389,025	6.12 × 10^−9^	8.21	Intergenic	T	C
	07	T	C	7,389,133	6.12 × 10^−9^	8.21	Intergenic	C	T
	04	A	C	214,280,150	8.99 × 10^−9^	8.05	Intergenic	A	C
	12	A	G	223,949,835	1.11 × 10^−8^	7.95	Intergenic	A	G
	07	C	A	233,954,670	1.12 × 10^−8^	7.95	Genic	C	A
Capsanthin	10	T	C	8,626,608	2.17 × 10^−7^	6.66	Intergenic	C	T
	02	C	T	136,572,333	2.49 × 10^−7^	6.60	Genic	C	T
	12	G	A	221,085,307	3.24 × 10^−7^	6.49	Genic	A	G
	06	G	A	240,758,527	6.70 × 10^−7^	6.17	Genic	A	G
	10	A	G	8,625,878	8.68 × 10^−7^	6.06	Intergenic	G	A
Zeaxanthin	03	T	C	273,399,119	2.99 × 10^−11^	10.52	Genic	C	T
	07	C	T	209,703,812	2.03 × 10^−10^	9.69	Intergenic	T	C
	01	C	C	13,088,352	2.29 × 10^−10^	9.64	Intergenic	T	C
	01	T	T	13,088,406	2.29 × 10^−10^	9.64	Intergenic	G	T
	03	T	C	250,691,656	2.29 × 10^−10^	9.64	Genic	C	T
	04	A	C	221,648,109	2.29 × 10^−10^	9.64	Genic	C	A
	04	G	A	232,075,190	2.29 × 10^−10^	9.64	Genic	A	G
	08	A	G	158,075,413	2.29 × 10^−10^	9.64	Intergenic	G	A
	09	A	C	124,336,652	2.29 × 10^−10^	9.64	Intergenic	C	A
	09	A	G	135,166,449	2.29 × 10^−10^	9.64	Genic	G	A
	03	T	G	31,436,193	1.10 × 10^−9^	8.96	Genic	G	T
	08	T	C	135,871,114	1.10 × 10^−9^	8.96	Genic	C	T
	08	G	A	135,910,583	1.10 × 10^−9^	8.96	Genic	A	G
	08	G	A	167,703,221	1.10 × 10^−9^	8.96	Genic	A	G
	08	T	A	167,703,365	1.10 × 10^−9^	8.96	Genic	A	T
	08	C	T	170,330,854	1.10 × 10^−9^	8.96	Genic	T	C
	09	A	C	144,268,010	1.10 × 10^−9^	8.96	Intergenic	C	A
	12	G	C	9,887,870	1.10 × 10^−9^	8.96	Genic	C	G
	12	T	C	10,065,787	1.10 × 10^−9^	8.96	Intergenic	C	T
	12	T	C	23,244,295	1.10 × 10^−9^	8.96	Intergenic	C	T
	12	T	A	10,062,899	4.11 × 10^−9^	8.39	Intergenic	A	T
β-Cryptoxanthin	03	A	G	50,199,834	2.41 × 10^−11^	10.62	Genic	G	A
	03	C	T	234,486,991	4.89 × 10^−8^	7.31	Intergenic	T	C
	06	C	T	222,803,463	1.08 × 10^−7^	6.97	Genic	T	C
α-Carotene	09	A	G	1,449,430	8.02 × 10^−7^	6.10	Genic	A	G
	12	C	T	221,317,241	9.17 × 10^−7^	6.04	genic	T	C
Dihydrocapsaicin	08	T	C	25,161,329	4.61 × 10^−9^	8.34	Intergenic	C	T
	01	A	G	208,666,749	5.36 × 10^−9^	8.27	Genic	G	A
	01	A	T	209,615,478	5.36 × 10^−9^	8.27	Genic	T	A
	01	G	A	214,441,815	5.36 × 10^−9^	8.27	Intergenic	A	G
	01	G	A	214,441,917	5.36 × 10^−9^	8.27	Intergenic	A	G
	01	C	T	214,442,584	5.36 × 10^−9^	8.27	Intergenic	T	C
	01	T	C	132,753,120	1.07 × 10^−8^	7.97	Intergenic	C	T
	01	C	A	176,184,702	1.72 × 10^−8^	7.77	Genic	A	C
	06	G	T	27,230,404	6.43 × 10^−8^	7.19	Genic	T	G
	07	A	C	6,571,083	9.69 × 10^−8^	7.01	Intergenic	C	A

**Table 3:** SNP markers showing pleiotropic effects among the different carotenoids.

Traits	Chr.	Allele	Positions (bp.)	−Log_10_ (*p*-Value)	Feature	Gene	Descriptions
Violaxanthin, antheraxanthin, capsorubin and capsanthin	Chr12	A/G	221,085,307	6.92	Genic	CA12g19380	6,7-dimethyl-8-ribityllumazine synthase
Violaxanthin and antheraxanthin	Chr12	T/C	221,105,042	6.01	Intergenic	-	-
Violaxanthin and capsorubin	Chr07	A/C	223,699,776	6.35	Intergenic	-	-
	Chr03	A/T	211,135,220	6.75	Genic	CA03g18160	4-hydroxycinnamoyl-CoA ligase 2
Capsorubin and capsanthin	Chr02	T/C	136,572,333	9.75	Genic	CA02g25010	Detected protein of unknown function

## Data Availability

The data used in this study are available in the manuscript and Supplementary File. For additional inquiries, please contact the corresponding author directly via email.

## Supplementary Materials

The following supporting information can be downloaded at: https://www.mdpi.com/article/10.3390/ijms241813885/s1.
